# Whole-Genome Sequencing of Extended-Spectrum β-Lactamase-Producing *Klebsiella pneumoniae* Isolated from Human Bloodstream Infections

**DOI:** 10.3390/pathogens14030205

**Published:** 2025-02-20

**Authors:** Carolina Sabença, Rani Rivière, Eliana Costa, Sara Sousa, Manuela Caniça, Vanessa Silva, Gilberto Igrejas, Carmen Torres, Patrícia Poeta

**Affiliations:** 1 MicroART-Antibiotic Resistance Team, Department of Veterinary Sciences, University of Trás-os-Montes and Alto Douro, 5000-801 Vila Real, Portugal; anacarolina@utad.pt; 2 Department of Genetics and Biotechnology, University of Trás-os-Montes and Alto Douro, 5000-801 Vila Real, Portugal; 3 Functional Genomics and Proteomics Unit, University of Trás-os-Montes and Alto Douro, 5000-801 Vila Real, Portugal; 4 Associated Laboratory for Green Chemistry (LAQV-REQUIMTE), University NOVA of Lisboa, 2829-516 Caparica, Portugal; 5 National Reference Laboratory of Antibiotic Resistances and Healthcare Associated Infections, Department of Infectious Diseases, National Institute of Health Dr. Ricardo Jorge, 1649-016 Lisbon, Portugal; 6 Hospital Centre of Trás-os-Montes and Alto Douro, Clinical Pathology Department, 5000-508 Vila Real, Portugal; 7 Centre for the Studies of Animal Science, Institute of Agrarian and Agri-Food Sciences and Technologies, University of Porto, 4051-401 Porto, Portugal; 8 Associate Laboratory for Animal and Veterinary Science (AL4AnimalS), 5000-801 Vila Real, Portugal; 9 Area Biochemistry and Molecular Biology, University of La Rioja, 26006 Logroño, Spain; 10 CECAV—Veterinary and Animal Research Centre, University of Trás-os-Montes and Alto Douro, 5000-801 Vila Real, Portugal

**Keywords:** extended-spectrum β-lactamases, *Klebsiella pneumoniae*, bloodstream infections, whole-genome sequencing

## Abstract

*Klebsiella pneumoniae* is a Gram-negative bacterium commonly associated with bloodstream infections (BSIs), which can lead to severe clinical outcomes, especially in immunocompromised individuals or patients with underlying health conditions. The increasing prevalence of *K. pneumoniae* that produces extended-spectrum β-lactamases (ESBL) poses a significant challenge for treatment and infection control, necessitating a swift diagnostic approach and tailored antimicrobial therapy to improve patient outcomes. A total of 32 *K. pneumoniae* isolates were recovered from BSIs from December 2021 to August 2022. Whole-genome sequencing (WGS) was performed on the 14 ESBL-producing isolates. All ESBL isolates carried the *bla*_CTX-M-15_ gene, together with other β-lactamase-encoding genes (*bla*_TEM-1_, *bla*_SHV-28_, *bla*_SHV-26_, or *bla*_OXA-1_). Three of the isolates also carried the *bla*_KPC-3_ gene. Resistance genes to quinolones, sulfonamides, tetracycline, aminoglycosides, and chloramphenicol were also detected. We can conclude that the presence of ESBL-producing isolates among *K. pneumoniae* of BSIs raises concerns, since these enzymes limit the available treatment options, and future research must include studies on alternative therapies for dealing with resistant bacterial infections and developing new approaches to disease treatment.

## 1. Introduction

In recent decades, antibiotic-resistant bacteria have become an urgent public health problem. This fact has increased attention to the extended-spectrum β-lactamase (ESBLs) group of enzymes in strains of the order Enterobacterales. These enzymes can be acquired via plasmid transfer, favoring the resistance of their host strains to β-lactam antibiotics and, in particular, the third-generation cephalosporins [1,2,3]. ESBLs are a diverse group of β-lactamases, and their classification is based on their substrate profile and genetic relatedness. The most common types of ESBLs include CTX-M enzymes, as well as some variants of TEM and SHV. The CTX-M enzymes, in particular, have emerged as the most prevalent in recent years, with the CTX-M-15 variant being widely distributed [4]. ESBLs confer resistance by hydrolyzing third-generation cephalosporins but generally do not affect carbapenems [5].

Strains producing ESBLs together with other mechanisms of resistance for non-beta-lactams have increased, what facilitate he use carbapenems for treatment. However, the emergence of carbapenemases remains a significant public health concern [6,7]. ESBL-encoding genes are often localized on mobile genetic elements, such as plasmids, facilitating horizontal gene transfer between bacterial genera and species [8]. This ability to spread across diverse bacterial species and genera complicates treatment and increases the likelihood of outbreaks. The transfer of these genes can occur within a single bacterial species and between Enterobacterales and non-Enterobacterales members, such as *Pseudomonas* or *Acinetobacter*, further exacerbating the challenge of multidrug resistance [9,10].

According to the 2023 annual epidemiological report from the European Centre for Disease Prevention and Control (ECDC) in Portugal, 50.7% of invasive *Klebsiella pneumoniae* isolates and 17.2% of *Escherichia coli* isolates were resistant to third-generation cephalosporins, while 13.1% of *K. pneumoniae* and 0.5% of *E. coli* were resistant to carbapenems, reaching higher values since 2006 for *K. pneumoniae* and 2001 for *E. coli* (https://atlas.ecdc.europa.eu/; accessed on 14 January 2025) [11]. The emergence and subsequent surveillance of strains producing ESBLs are necessary for good hospital management and patient therapy, particularly for those with a clinical diagnosis of septicemia.

*Klebsiella pneumoniae* is one of the species that have been most frequently isolated from clinical specimens and is a cause of sepsis, meningitis, and bacteremia. Many *K. pneumoniae* strains are resistant to a broad set of antimicrobial drugs, and their capacity to produce ESBLs is an essential mechanism of bacterial resistance, which increases in both the hospital and community environment [12,13,14].

Two previous studies have been conducted in a hospital located in North Portugal, which showed the predominance of CTX-M-15 among ESBL-producing *K. pneumoniae* isolates alongside SHV-type β-lactamases like SHV-27 and SHV-12 in isolates obtained during 2016–2018 from different origins, including bloodstream infections (BSIs) [15,16]. Both studies reported carbapenem-resistant isolates, and some *K. pneumoniae* strains showed a cooccurrence of ESBL and carbapenemase-encoding genes, highlighting the alarming levels of multidrug resistance within this species [15,16]. A recent study was also performed in the same hospital with *K. pneumoniae* isolates recovered from different origins during a nine-month period in 2021–2022, analyzing their content in β-lactamases by PCR [17]. The present study is focused on the characterization by whole-genome sequencing (WGS) of a collection of 14 ESBL-producing *K. pneumoniae* isolates recovered from BSIs during 2021–2022 in the same hospital in order to deepen in the molecular characterization of *K. pneumoniae* isolates that circulate in this hospital in invasive infections.

## 2. Materials and Methods

### 2.1. Bacterial Isolates and Identification

Thirty-two *K. pneumoniae* isolates were recovered from BSIs in the Hospital Center of Trás-os-Montes and Alto Douro between December 2021 and August 2022 and were identified by VITEK^®^ 2 Compact (BioMérieux, Auvergne-Rhône-Alpes, France). Fourteen of these isolates were ESBL producers (43.7%) and were selected for further characterization by WGS. The type of β-lactamases of two of these 14 ESBL-producer strains were already studied by PCR in a previous study [17].

### 2.2. Whole-Genome Sequencing of ESBL-Producing Isolates

WGS was performed in the 14 ESBL-producing *K. pneumoniae* isolates recovered from BSIs in the studied period. Initially, the strains were seeded in the differential medium, MacConkey, and isolated in nutrient agar. According to the manufacturer’s protocol, the genomic DNA was extracted using the Magna Pure 96 system (Roche, Basel, Switzerland). The DNA concentration was measured using a QubitTM 4 fluorometer (Thermo Scientific, Waltham, MA, USA). Sequencing libraries were prepared using the Nextera XT library preparation kit (Illumina, San Diego, CA, USA) and sequenced using Illumina MiSeq with 150 bp paired-end reads. Raw reads were then submitted for bioinformatics analysis using multiple tools to process the WGS data. De novo assembly, species confirmation, and sequence-type raw data quality control were performed using INNUca (v 4.2.2-02) (https://github.com/B-UMMI/INNUca accessed on 23 September 2024). The assessment of read quality, trimming, and estimated genome completeness were performed using FastQC (v 0.11.5) (http://www.bioinformatics.babraham.ac.uk/projects/fastqc/; accessed on 23 September 2024), Trimmomatic (v 0.38) [18], and BUSCO (v 5.5.0_cv1) (https://gitlab.com/ezlab/busco#how-to-cite-busco; accessed on 23 September 2024), respectively. The identification of the species was confirmed by calculating the average nucleotide identity (ANI) using FastANI (v 1.33) [19], where the query genome is compared against the complete assembled reference genomes downloaded from the NCBI GenBank database (https://www.ncbi.nlm.nih.gov/datasets/genome/?taxon=570; accessed on 23 September 2024). For ARGs detection, abriTAMR (v 1.0.14) and ABRicate (v 1.0.1) were used (http://github.com/tseemann/abricate; accessed on 23 September 2024) [20]. In ABRicate, the following public databases were used: argannot, resfinder, ncbi, card, plasmidfinder, and vfdb. Multilocus Sequence Typing (MLST) was employed to categorize the isolates into sequence types.

## 3. Results

### 3.1. Genomic Characterization by Whole-Genome Sequencing

#### 3.1.1. β-Lactamase and Other Antimicrobial Resistance Genes

The analysis of the genomes of the 14 ESBL-producing *K. pneumoniae* isolates allows us to deepen in the molecular characteristics of these isolates. Three sequence types (ST) were identified in these 14 ESBL-positive isolates: ST15 (8 isolates), ST307 (4 isolates), and ST34 (2 isolates) (Table 1). The *bla*_CTX-M-15_ gene was found in all 14 isolates, and other β-lactamases genes were also identified, such as *bla*_TEM-1_, *bla*_SHV-28_, *bla*_SHV-26_, and *bla*_OXA-1_. Moreover, the *bla*_KPC-3_ gene was identified in three of these isolates that also showed imipenem resistance (Table 1). Regarding other resistance genes, it was found that the *aac*(3)-IIe, *aph*(3″)-Ib, *aph*(6)-Id, and *aad*A16 genes confer resistance to aminoglycosides, as well as the *aac*(6′)-Ib-cr gene that confers simultaneous resistance to aminoglycoside and fluoroquinolone antibiotics. Concerning resistance to fluoroquinolone antibiotics, the *qnr*B1 and *qnr*B6 genes were detected, but efflux pump conferring resistance to this family of antibiotics, such as *oqx*A, *oqx*A10, *oqx*B19, and *oqx*B20, was also identified. The *sul*1, *sul*2, *dfr*A14, and *dfr*A27 genes were detected, which confer resistance to trimethoprim-sulfamethoxazole. The *tet*D gene, responsible for conferring resistance to tetracycline, was the only associated gene identified. Lastly, three different genes were found to confer resistance to chloramphenicol: *cat*A1, *cat*A2, and *cat*B3 (Table 1).

#### 3.1.2. Plasmid Replicons

Both an incompatibility (Inc) group and colicinogenic (Col) plasmids were detected in the genomes of the 14 sequenced *K. pneumoniae* isolates. Inc plasmids were present in all isolates; however, Col plasmids were only detected in 7 isolates. The most detected plasmid was IncFIB(K) (n = 14), followed by IncFIA(HI1) (n = 13) and IncR (n = 12). Among the Col plasmids, the most frequently detected was Col440I, present in six isolates. In two isolates, we identified two copies of the IncFII and ColRNAI plasmids (Table 1).

#### 3.1.3. Virulence Genes

The 14 sequenced genomes were screened for genes related to virulence factors. Consequently, the *ybt*P and *ybt*Q virulence genes were detected in six isolates. Also, we detected the *tsh* virulence gene in one isolate, which encodes temperature-sensitive hemagglutinin. We similarly detected other genes that are not virulence genes but may have a role in helping bacterial survival, such as ferrous iron efflux (*fie*F), and eight heat resistance genes (*hsp*20, *psi*-GI, *kef*B-GI, *trx*LHR, *hde*D-GI, *yfd*X1, *yfd*X2, and *shs*P) (Table 1).

## 4. Discussion

The purpose of the present study was to analyze the WGS of the ESBL-producing *K. pneumoniae* isolates recovered at the hospital level (hospital center of Trás-os-Montes and Alto Douro) of BSIs during a period of nine months. *K. pneumoniae* is frequently resistant to a wide range of antibiotics, including different β-lactams by β-lactamases [21]. In detail, infections caused by ESBL-producing Enterobacterales represent a worldwide issue concerning public health, especially given their association with poor outcomes, growing community-onset, and high ecological treatment costs [22]. Although the ESBL family is heterogeneous, the global pandemic of plasmids carrying CTX-M-type genes, which started mainly in the 2000s, is the primary driver of ESBL dissemination and has replaced other ESBL enzymes [23].

For this study, 14 ESBL-producing *K. pneumoniae* isolates recovered from BSIs from a Portuguese hospital were analyzed. One previous study was conducted on *K. pneumoniae* isolates of BSIs in the same hospital, but the study was performed four years before [15], and another one was conducted in isolates from the same period as the present study [17]. However, neither of these studies included the characterization of isolates by WGS. Concerning *K. pneumoniae* BSIs and the significant impact of the latter infections in the clinical setting drove our interest in studying these infections. BSIs due to *K. pneumoniae* are associated with higher mortality rates than infections at other body sites [24]. Consequently, filling this gap in the literature is imperative to improve patient outcomes and provide evidence-based treatment options for such serious infections. A study by Gorrie et al. conducted a year-long prospective surveillance study of *K. pneumoniae* clinical isolates in hospital patients and reported that 10% of patients had *K. pneumoniae* isolated from disseminated infections (bloodstream and/or cerebral spinal fluid), a similar percentage to ours [25]. This consistency across studies suggests that a range of infections caused by *K. pneumoniae* progress to BSIs, suggesting that bacterial or host factors may contribute to the potential to disseminate *K. pneumoniae*.

Regarding ESBL production, the reported ESBL production rates in BSIs caused by Enterobacterales are highly variable. The European Centre for Disease Prevention and Control (ECDC), in its 2023 annual epidemiological report, revealed that, in Portugal, the percentage of invasive *K. pneumoniae* isolates resistant to third-generation cephalosporins was 50.7% (https://atlas.ecdc.europa.eu/; accessed on 14 January 2025) [11]. This ECDC value represents the highest recorded percentage for third-generation cephalosporins since surveillance began in 2006 in Portugal and is a little higher than the one found in this study (43.7%). This upward trend in resistance poses a substantial challenge to public health, as it limits the effectiveness of commonly used antibiotics and complicates treatment options for infections caused by *K. pneumoniae* [26]. In Ethiopia, 67.3% of BSI isolates were ESBL producers [27]. A study conducted in Egypt reported lower percentages of ESBL-producing isolates than the previous study at 48.93% [28]. Also, a study in India demonstrated that 43.5% of Gram-negative isolates were β-lactamase producers [29]. The high prevalence of CTX-M among *K. pneumoniae* isolates is an important finding for applying rapid diagnostic methods. For example, rapid immunochromatographic or molecular methods capable of detecting CTX-M could be implemented in areas with a high prevalence of CTX-M ESBL [30]. These rates of ESBL occurrence represent a worryingly high prevalence amongst BSIs. This paper also underlines once more the call to strengthen surveillance and detection mechanisms in hospitals towards BSIs caused by ESBL-producing Enterobacterales.

Concerning the analysis of the whole genomes, three different sequence types were detected among the 14 ESBL-producing *K. pneumoniae* sequenced isolates: ST15 (57.1%), ST307 (28.6%), and ST34 (14.3%). Various studies have described different STs among the BSI *K. pneumoniae* isolates, where ST15 frequently emerges alongside other common types, such as ST11 and ST23 [31,32,33,34]. The prominence of ST15 in our isolates is notable, as it is associated with multidrug resistance and often implicated in healthcare-associated infections, indicating a potentially high transmission and adaptation rate in clinical settings. However, less commonly found, both ST34 and ST307 have also been reported [35,36,37,38]. The identification of these sequence types in our study highlights the diversity and adaptability of *K. pneumoniae* strains contributing to BSIs.

Regarding β-lactamase genes, we verified a predominance of the *bla*_CTX-M-15_ gene, which was also predominant in the previous studies conducted by our group [15]. Still, in some strains, this gene was associated with a carbapenemase gene (*bla*_KPC_ or *bla*_OXA-48_). It is interesting to show that, in four years, the genetic lineages seemed to differ in both studies. While our study reported isolates belonging to ST15, ST307, and ST34, the previous one reported isolates belonging to ST348, ST11, and ST15. ESBLs encoded by *bla*_CTX-M-15_ are one of the most commonly found among *K. pneumoniae* isolates causing BSIs. Multiple studies have reported a high prevalence of this gene; for example, in South Korea, a study conducted between 2017 and 2019 verified that the dominant ESBL type in *K. pneumoniae* blood isolates was CTX-M-group 1 (78.5%), which included CTX-M-15 (n = 407) and CTX-M-55 (n = 5), while CTX-M-group 9 was less frequently identified (14.0%) but included CTX-M-14 (n = 60) and CTX-M-27 (n = 8) [39]. Also, in Italy, among the *K. pneumoniae* isolates, all encoded CTX-M-15 [40], and in Algeria, CTX-M-15 ESBL-*K. pneumoniae* strains infected 8.2% of neonates [41]. Regarding carbapenemase-producing enzymes, we detected the *bla*_KPC-3_ gene (in addition to *bla*_CTX-M-15_) in three isolates analyzed by WGS. The *bla*_KPC_ gene is frequent in BSI isolates of *K. pneumoniae*. In this sense, a prevalence of this gene of 33.3% has been identified in China [42] and 67.4% in carbapenem-resistant Enterobacterales isolates in India [43]. In Brazil, the prevalence of the *bla*_KPC_ gene in carbapenem-resistant *K. pneumoniae* BSI isolates was 90.4% [44]. It has been shown that the presence of *bla*_KPC_ genes increases the total mortality in BSI patients [42,44]. In the case of Enterobacterales, KPC-2 and -3 are the most commonly detected variants [35,39,45,46]. In Portugal, CTX-M-15 and KPC-3 are predominant, which is in line with our results. In the last five years, multiple studies have reported the presence of these enzymes, in particular KPC-3, among Enterobacterales isolates, underscoring their significance in antimicrobial resistance profiles within the region [15,16,47,48,49,50,51,52]. However, the genetic lineages identified in our study appear to diverge from those previously reported in Portugal. While ST15 is frequently encountered in Portuguese clinical bacterial isolates, the other two sequence types have been less commonly documented in healthcare settings [15,16,48,50,52,53,54,55]. The persistence of these resistance mechanisms reflects local and global trends in antimicrobial resistance and highlights the need for ongoing surveillance and stringent infection control measures.

Besides ESBLs, several other AMR genes were identified in BSI *K. pneumoniae* isolates, including those conferring resistance to aminoglycoside, fluoroquinolone, trimethoprim-sulfamethoxazole, tetracycline, and chloramphenicol. Among the commonly detected genes, those that encode aminoglycoside resistance included *aac*(3)-IIe, *aph*(3″)-Ib, *aph*(6)-Id, and *aad*A16; fluoroquinolones resistance included *qnr*B1, *qnr*B6, and co-resistance to fluoroquinolones *aac*(6′)-Ib-cr genes; trimethoprim-sulfamethoxazole included *sul*1, *sul*2, *dfr*A14, and *dfr*A27; tetracyclines included *tet*D genes; and chloramphenicol included *cat*A1, *cat*A2, and *cat*B3. Also, genes for the efflux pumps, such as *oqx*A, *oqx*A10, *oqx*B19, and *oqx*B20, which further facilitated the ability of bacteria to efflux a wide range of antibiotics, were detected. Other studies have also identified several genes that confer resistance to these antibiotic classes, for example, a study conducted in the Eastern Democratic Republic of Congo reported similar results by finding that the *K. pneumoniae* isolates carried at least one gene linked to resistance to aminoglycosides and/or quinolones genes (*aac*(3)-IId, *aac*(3)-IIe, *aad*A1, *aad*A16, *aad*A2, *aph*(3′)-Ia, *aph*(3″)-Ib, *aph*(6)-Id, *aac*(6′)-Ib-cr, or *aac*(6′)-Ib-cr5), as well as to phenicols (*cat*A1, *cat*A2, and *cat*B); sulfonamides (*sul*1 and *sul*2); tetracycline (*tet*A or *tet*D); trimethoprim (*dfr*A1, *dfr*A12, *dfr*A14, *dfr*A15, *dfr*A25, and *dfr*A27); plasmid-mediated quinolone resistance genes (*qnr*B, *qnr*B1, *qnr*B2, *qnr*B6, and *qnr*S1); and also efflux pump genes (*oqx*A, *oqx*B, *oxq*B5, *oqx*B19, *oqx*B21, and/or *oqx*B32) [56]. The diverse AMR genes that underpin its complex resistance mechanisms stress the need for intensive surveillance and targeted antimicrobial strategies in controlling this pathogen.

Regarding detected plasmids, we have identified either Inc plasmids or Col plasmids. Among the Inc plasmids, IncFIB(K), IncFIA(HI1), and IncR were the most detected ones, and regarding Col plasmids, we have detected Col440I, Col440II, and ColRNAI. Plasmid incompatibility groups, especially Inc-type plasmids, are known to play a significant role in the spread of antimicrobial resistance genes. IncF and IncR plasmids, for example, are frequently associated with resistance genes and have been widely documented in *K. pneumoniae* BSI isolates [57]. The high prevalence of IncFIB(K) and IncR plasmids is consistent with reports linking these plasmids to multidrug-resistant strains and their capacity to carry multiple resistance genes, thus enhancing the potential for resistance dissemination within hospital settings. While Inc plasmids are generally more common than Col plasmids in *K. pneumoniae*, Col-type plasmids like Col440I and ColRNAI can also contribute to survival under antibiotic pressure by facilitating resistance gene transfer, albeit on a smaller scale [58,59,60,61]. Their presence suggests that Col plasmids might play a secondary yet complementary role in the persistence and adaptability of *K. pneumoniae* in clinical environments. Understanding the roles these plasmids play in resistance gene transmission is crucial, as they represent key targets for surveillance and control measures aimed at reducing the spread of resistant strains in healthcare settings.

Regarding virulence genes, the detection of *ybt*P and *ybt*Q virulence genes was made in six isolates. These genes belong to the *ybt*PQXS operon, making part of a yersiniabactin biosynthesis and transport system. This operon is critical for acquiring and transporting iron through a siderophore-mediated mechanism, where yersiniabactin acts as the siderophore [62,63,64]. We also detected the temperature-sensitive hemagglutinin gene (*tsh*) in one isolate. This virulence factor promotes adherence to host cells, a critical first step in colonization and infection, and degrades the host proteins, which may facilitate tissue invasion and nutrient acquisition [65,66]. Apart from the virulence-associated genes identified, we also found in our isolates other genes that are not categorized under the traditional virulence factors but may considerably enable bacteria to survive against various environmental stresses. For instance, the product of the *fie*F gene is a ferrous iron efflux pump that helps bacteria regulate and export excessive iron ions. This function helps reduce metal-induced stress, supporting bacterial growth and contributing to the bacteria’s ability to survive in metal-rich environments, including during host infection [67,68]. We also found eight genes related to heat resistance: *hsp*20, *psi*-GI, *kef*B-GI, *trx*LHR, *hde*D-GI, *yfd*X1, *yfd*X2, and *shs*P. These same eight genes have also been reported in a study performed on *E. coli* from cattle [69]. These heat resistance genes are crucial factors for bacterial survival in extreme environments. For example, the *hsp*20 gene encodes for a small-sized heat shock protein that protects bacterial cells under various stressful conditions, such as heat shock, osmotic shock, and oxidative stress [70,71]. Another example is the locus of heat resistance (LHR), a genomic island that confers exceptional heat resistance in Enterobacterales, including *E. coli* and *Salmonella*. It includes genes such as *yfd*X1, *yfd*X2, and *hde*D, which are necessary for high-level heat resistance [72,73,74]. Altogether, these genes confer an impressive armamentarium on bacteria to resist stressing conditions and raise their survival capability outside their host and in environmental reservoirs. This indirectly contributes to the persistence and spread of antibiotic-resistant bacteria, as such genes support their populations in persisting and expanding under the variability of environmental conditions, challenges by antibiotics, or host immune defenses. These survival genes are important in understanding bacterial resilience, though virulence-related, because they can enable the long-term persistence of pathogenic and antibiotic-resistant strains in the ecosystem.

## 5. Conclusions

Conclusively, the results of this research paper highlight important microbiological aspects of BSIs due to ESBL-producing *K. pneumoniae* in a Portuguese hospital. Crucial findings from this study include ESBL production, with most strains carrying CTX-M-15 as their major ESBL genotype. Further significant aspects involved the identification of the *bla*_KPC-3_ carbapenemase gene. High levels of ESBL-producing isolates raise concerns, since these enzymes limit the available treatment options. To this end, future research must include studies on alternative therapies for dealing with resistant bacterial infections and the development of new approaches to disease treatment. This also calls for the continuous monitoring of resistance patterns and emergence of new mechanisms, provision of point-of-care tests for better management of infections through correct drug application, more stringent efforts related to infection control and the prevention of resistant bacteria spreading, and finally, prompt action taken by every institution towards the optimal use of antibiotics due to global changes in resistance levels that are impermanent and the coexistence of which, with a watchful selection of antimicrobials, is imperative to retaining all available options for the treatment and management of severe infections. A combined strategy of surveillance, hygienic practices, diagnostics, and antibiotic optimization programs is necessary to combat the menace presented by antibiotic-resistant *K. pneumoniae*.

## Figures and Tables

**Table 1:** Molecular characterization of 14 ESBL-producing *K. pneumoniae* isolates of BSIs.

Isolate	MLST	β-Lactamase Genes	Carbapenemase Genes	Other Resistance Genes	Plasmids	Virulence Genes and Others
HS107	ST15	*bla*_CTX-M-15_, *bla*_TEM-1_, *bla*_SHV-28_, *bla*_OXA-1_	*bla*_KPC-3_	*aac*(3)-IIe, *aph*(3″)-Ib, *aph*(6)-Id, *aac*(6′)-Ib-cr, *qnr*B1, *oqx*A, *oqx*B20, *sul*2, *dfr*A14, *cat*B3	IncFII, IncFIA(HI1), IncFIB(K), IncR, Col440I	*ybt*P, *ybt*Q, *fie*F, *hsp*20
HS119	ST15	*bla*_CTX-M-15_, *bla*_TEM_, *bla*_SHV-28_, *bla*_OXA-1_	-	*aac*(3)-IIe, *aph*(3″)-Ib, *aph*(6)-Id, *aac*(6′)-Ib-cr, *qnr*B1, *oqx*A, *oqx*B20, *sul*2, *dfr*A14, *cat*A1, *cat*B3	IncFIA(HI1), IncFIB(K), IncR, Col440I Col(MG828)	*ybt*P, *ybt*Q, *fie*F, *hsp*20
HS143	ST15	*bla*_CTX-M-15_, *bla*_TEM-1_, *bla*_SHV-28_, *bla*_OXA-1_	-	*aac*(3)-IIe, *aph*(3″)-Ib, *aph*(6)-Id, *aac*(6′)-Ib-cr, *qnr*B1, *oqx*A, *oqx*B20, *sul*2, *dfrA*14, *cat*A1, *cat*B3,	IncFII, IncFIA(HI1), IncFIB(K), IncR, Col440I	*ybt*P, *ybt*Q, *fie*F, *hsp*20
HS203	ST15	*bla*_CTX-M-15_, *bla*_TEM-1_, *bla*_SHV-28_, *bla*_OXA-1_	-	aac(3)-IIe, *aph*(3″)-Ib, *aph*(6)-Id, *aac*(6′)-Ib-cr, *qnr*B1, *oqx*A, *oqx*B20, *sul*2, *dfr*A14, *cat*A1, catB3	IncFII, IncFIA(HI1), IncFIB(K), IncR	*ybt*P, *ybt*Q, *fie*F, *hsp*20
HS255	ST15	*bla*_CTX-M-15_, *bla*_TEM_, *bla*_SHV-28_, *bla*_OXA-1_	*bla*_KPC-3_	*aac*(3)-IIe, *aph*(3″)-Ib, *aph*(6)-Id, *aac*(6′)-Ib-cr, *qnr*B1, *oqx*A, *oqx*B20, *sul*2, *dfr*A14, *cat*A1, *cat*B3	IncFII, IncFIA(HI1), IncFIB(K), IncR, Col440I	*ybt*P, *ybt*Q, tsh, *fie*F, *hsp*20
HS265	ST307	*bla*_CTX-M-15_, *bla*_TEM-1_	-	*aac*(3)-IIe, *aad*A16, *aac*(6′)-Ib-cr, *qnr*B6, *sul*1, *dfr*A27, *tet*D, *cat*A2	IncFIA(HI1), IncFIB(K), IncR	*fie*F, *hsp*20
HS269	ST307	*bla*_CTX-M-15_, *bla*_TEM-1_, *bla*_SHV-28_	-	*aac*(3)-IIe, *aad*A16, *aac*(6′)-Ib-cr, *qnr*B6, *sul*1, *dfr*A27, *tet*D, *cat*A2	IncFIA(HI1), IncFIB(K), IncR	*fie*F, *hsp*20
HS286	ST34	*bla*_CTX-M-15_, *bla*_TEM-1_, *bla*_SHV-26_, *bla*_OXA-1_	-	*aac*(3)-IIe, *aph*(3″)-Ib, *aph*(6)-Id, *aac*(6′)-Ib-cr, *qnr*B1, *oqx*A10, *oqx*B19, *sul*2, *dfr*A14, *cat*B3	IncFII *, IncFIA(HI1), IncFIB(K), ColRNAI	*fie*F, *hsp*20, *shs*P, *yfd*X1, *yfd*X2, *hde*D-GI, *trx*LHR, *ke*fB-GI, *psi*-GI
HS289	ST307	*bla*_CTX-M-15_, *bla*_SHV-28_	-	*aad*A16, *aac*(6′)-Ib-cr, *qnr*B6, *dfr*A27, *tet*D, *cat*A2	IncFIA(HI1), IncFIB(K), IncR	*fie*F, *hsp*20
HS290	ST15	*bla*_CTX-M-15_, *bla*_TEM-1_, *bla*_SHV-28_, *bla*_OXA-1_	*bla*_KPC-3_	*aac*(3)-IIe, *aph*(3″)-Ib, *aph*(6)-Id, *aac*(6′)-Ib-cr, *oqx*A, *oqx*B20, *sul*2, *dfr*A14, *cat*B3	IncFII, IncFIB(K), IncR, Col440I	*ybt*P, *ybt*Q, *fie*F, *hsp*20
HS292	ST307	*bla*_CTX-M-15_, *bla*_SHV-28_	-	*aad*A16, *aac*(6′)-Ib-cr, *qnr*B6, *dfr*A27, *tet*D, *cat*A2	IncFIA(HI1), IncFIB(K), IncR	*fie*F, *hsp*20
HS302	ST15	*bla*_CTX-M-15_, *bla*_TEM-1_, *bla*_SHV-28_	-	*aac*(3)-IIe, *aph*(3″)-Ib, *aph*(6)-Id, *qnr*B1, *oqx*A, *oqx*B20, *sul*2, *dfr*A14	IncFII, IncFIA(HI1), IncFIB(K), IncFIB(pKPHS1), IncR	*fie*F, *hsp*20
HS321	ST15	*bla*_CTX-M-15_, *bla*_TEM-1_, *bla*_SHV-28_	-	*aac*(3)-IIe, *aph*(3″)-Ib, *aph*(6)-Id, *sul*2, *qnr*B1, *oqx*A *oqx*B20, *dfr*A14	IncFII, IncFIA(HI1), IncFIB(K), IncFIB(pKPHS1), IncR	*fie*F, *hsp*20
HS331	ST34	*bla*_CTX-M-15_, *bla*_TEM-1_, *bla*_SHV-26_, *bla*_OXA-1_	-	*aac*(3)-IIe, *aph*(3″)-Ib, *aph*(6)-Id, *aac*(6′)-Ib-cr, *qnr*B1, *oqx*A10, *oqx*B19, *sul*2, *dfr*A14, *cat*B3	IncFII *, IncFIA(HI1), IncFIB(K), Col440I, Col440II, ColRNAI *	*fie*F, *hsp*20, *shs*P, *yfd*X1, *yfd*X2, *hde*D-GI, *trx*LHR, *ke*fB-GI, *psi*-GI

* Two copies were detected.

## Data Availability

The authors confirm that the data supporting the findings of this study are available within the article.
